# Corrigendum: Identification of Immune-Related Prognostic Genes and LncRNAs Biomarkers Associated With Osteosarcoma Microenvironment

**DOI:** 10.3389/fonc.2020.620320

**Published:** 2020-11-30

**Authors:** Tao Zhang, Yingli Nie, Haifa Xia, Yanbin Zhang, Kailin Cai, Xiangdong Chen, Huili Li, Jiliang Wang

**Affiliations:** ^1^Department of Anesthesiology, Union Hospital, Tongji Medical College, Huazhong University of Science and Technology, Wuhan, China; ^2^Department of Dermatology, Wuhan Children’s Hospital (Wuhan Maternal and Child Healthcare Hospital), Tongji Medical College, Huazhong University of Science and Technology, Wuhan, China; ^3^Department of Orthopaedics, Union Hospital, Tongji Medical College, Huazhong University of Science and Technology, Wuhan, China; ^4^Department of Gastrointestinal Surgery, Union Hospital, Tongji Medical College, Huazhong University of Science and Technology, Wuhan, China

**Keywords:** osteosarcoma, immune, prognosis, biomarker, tumor microenvironment

In the original article, there was a mistake in the legend for **Figure 7D** as published. There was no Kaplan–Meier survival analysis of infiltrating Mast cells activated in **Figure 7**. The correct legend appears below.

**Figure 7:**
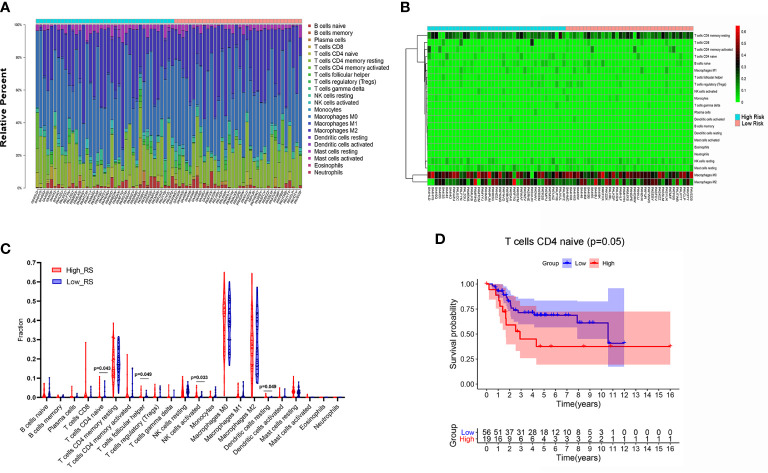
The composition **(A)** and heat map **(B)** of immune cells estimated by CIBERSORT algorithm in OSs. **(C)** The comparison of the fractions of immune cells between high- and low-risk group. **(D)** Kaplan–Meier survival analysis of overall survival between high and low level of infiltrating T-cell CD_4_ naive.

There was also an error in the format of author affiliations. Instead of:

“^1^Department of Anesthesiology, Tongji Medical College, Union Hospital, Huazhong University of Science and Technology, Wuhan, China

^2^Department of Dermatology, Tongji Medical College, Wuhan Children’s Hospital (Wuhan Maternal and Child Healthcare Hospital), Huazhong University of Science and Technology, Wuhan, China

^3^Department of Orthopaedics, Tongji Medical College, Union Hospital, Huazhong University of Science and Technology, Wuhan, China

^4^Department of Gastrointestinal Surgery, Tongji Medical College, Union Hospital, Huazhong University of Science and Technology, Wuhan, China”

it should be:

“^1^Department of Anesthesiology, Union Hospital, Tongji Medical College, Huazhong University of Science and Technology, Wuhan, China

^2^Department of Dermatology, Wuhan Children’s Hospital (Wuhan Maternal and Child Healthcare Hospital), Tongji Medical College, Huazhong University of Science and Technology, Wuhan, China

^3^Department of Orthopaedics, Union Hospital, Tongji Medical College, Huazhong University of Science and Technology, Wuhan, China

^4^Department of Gastrointestinal Surgery, Union Hospital, Tongji Medical College, Huazhong University of Science and Technology, Wuhan, China”.

The authors apologize for these errors and state that these do not change the scientific conclusions of the article in any way. The original article has been updated.

